# Dysfunctional Dynamics of Intra- and Inter-network Connectivity in Dementia With Lewy Bodies

**DOI:** 10.3389/fneur.2019.01265

**Published:** 2019-12-03

**Authors:** Wen-Ying Ma, Qun Yao, Guan-jie Hu, Chao-Yong Xiao, Jing-Ping Shi, Jiu Chen

**Affiliations:** ^1^Department of Neurology, The Affiliated Brain Hospital of Nanjing Medical University, Nanjing, China; ^2^Department of Neurosurgery, The Affiliated Brain Hospital of Nanjing Medical University, Nanjing, China; ^3^Department of Radiology, The Affiliated Brain Hospital of Nanjing Medical University, Nanjing, China; ^4^Institute of Brain Functional Imaging, Nanjing Medical University, Nanjing, China; ^5^Institute of Neuropsychiatry, The Affiliated Brain Hospital of Nanjing Medical University, Nanjing, China

**Keywords:** dementia with Lewy bodies, dynamic functional connectivity, *k*-means clustering, sliding-window, local efficiency

## Abstract

Dementia with Lewy bodies (DLB) is characterized by the transient fluctuating cognition and recurrent visual hallucinations, which may be caused by disorders of the intrinsic brain network dynamics. However, little is known regarding the dynamic features of the brain network behind these symptoms in DLB. In the present study, the intra- and inter-brain network dynamics were explored on a time scale in 17 DLB and 20 healthy controls (HC) applying a sliding-window method followed by *k*-means clustering analysis. To further evaluate the impact of network dynamics on brain performance, the local and global efficiency of the brain network was calculated. Compared with HC, the dynamic functional connectivity variation matrix in DLB patients was represented by a mixed change of intra-network increase and inter-network decrease. DLB patients devoted more time to a negative connectivity pattern, which represents a state of functional separation. Furthermore, the local efficiency of DLB patients was significantly lower compared with HC. These observations indicate an altered dynamic variability and disorders to the time allocation of state sequences in DLB, which might result in a disturbance of the intricate brain network dynamic properties, thereby leading to a lack of integration and flexibility and an ineffective brain function. In conclusion, dynamic functional connectivity analysis could identify differences between DLB and HC, providing evidences for DLB diagnosis and contributing to the understanding of the widespread clinical features and complex treatment strategies in DLB patients.

## Introduction

Dementia with Lewy bodies (DLB) is a growing concern worldwide, accounting for up to 15% of dementia cases (1). It is characterized by four core clinical features: fluctuating cognition, recurrent visual hallucinations, rapid eye movement sleep behavior disorder, and spontaneous parkinsonism (2). Multiple magnetic resonance imaging (MRI) studies revealed that the dysfunction of several brain connection networks is pertinent to the impairments in DLB. Fluctuating cognition and recurrent hallucinations, indicating transient abnormal brain functions, suggest that the connection disorders among brain's intrinsic networks may be dynamic. Studies on DLB, especially regarding fluctuation and recurrence, should focus more on the dynamic brain network properties enabled by time courses for a better understanding of its characteristics.

Recently, resting-state functional MRI (rs-fMRI) has become of increasing popularity in the exploration of the pathophysiological DLB mechanism employing various means. For instance, some studies delineated local functional connectivity (FC) patterns using seed region analysis, and found that the connectivity of default mode network (DMN), frontal-parietal network (FPN), sensorimotor network (SMN)-related brain regions (3, 4), and visual network (VIS) (5) of DLB were disrupted. In addition, functional brain network analysis based on independent component analysis (ICA) technique highlighted some disconnections within and between specific resting-state networks (RSNs), including DMN (6), SMN (7), and FPN (8), and the correlation between some connections and the frequency and severity of cognitive fluctuations. Furthermore, Peraza et al. (9) revealed altered network connectivity accompanied with frontal, parietal, and occipital lobes lower clustering coefficient in DLB patients by the graph theory, quantifying abnormal network characteristics. Generally, these findings provide an evidence regarding differences in functional brain network connectivity between DLB individuals and Parkinson's disease dementia or Alzheimer's disease, implying that the alterations in brain network organization play a potentially important role in comprehending the etiology of the core symptoms in DLB.

Nevertheless, static correlation between intra- and inter-networks is based only on the hypothesis that FC activity among different brain areas remains constant throughout the whole scanning process and the conventional methods mentioned above use static correlation (10, 11). However, several findings reported that the FC of human brain is not invariable (12, 13), showing a certain dynamic as time goes on, regardless of whether it is in the task state or under anesthesia (14). Since DLB is characterized by fluctuant and recurrent clinical symptoms, static functional measures cannot completely reflect the dynamic neural activity; thus, these traditional methods have recently faced challenges (14, 15). Dynamic functional connectivity (DFC) brain network analysis using the sliding-window method has been used to evaluate the relevant FC variations directly by dividing the whole scanning time series into many segments of the same size and observe the evolution of FC over time. This method offers a promising way for assessing the dynamic temporal organization of the resting state brain activity and brings fresh insights into the brain network fluctuation caused by brain disorders. Therefore, a great number of neuropsychiatric studies on major depressive disorder (16), schizophrenia (17, 18), epilepsy (19), and mild cognitive impairment (20), have been carried out. Despite the fact that DFC measurements have been increasingly used to investigate the way the brain is affected by the disease, neuroimaging investigations of DFC in DLB patients remain scarce. A recent work (21) applied the Product Hidden Markov Models (HMM) to compare the DFC changes between DLB and healthy participants, and a set of visual-related RSNs and FPN have been identified. However, this work mainly focused on the Product HMM from a modeling perspective, and less attention was paid on exploring the pathological mechanism of this disease. Thus, further investigations are needed to reveal the DFC characteristics of DLB, to deepen our knowledge in the underlying etiology, therefore providing important clues on the potential biomarkers for clinical diagnosis and treatment.

Hence, in the present study, sliding-window DFC analysis and exploratory *k*-means clustering technique were employed to systematically evaluate FC pattern dynamics in DLB patients. Our aim was to evaluate if the variability of DFC in DLB was significantly changed compared with HC both intra- and inter-network. In addition, a further aim considering the cognitive impairment was to evaluate if the temporal distribution among states of DLB subjects was aberrant, showing a less flexible state transition.

## Materials and Methods

### Participants and Assessment

A total of 17 DLB patients (mean age, 71.88 ± 5.69 years; range, 60–82 years; 13 male) and 20 healthy subjects (mean age, 67.80 ± 7.19 years; range, 56–77 years; 9 male) as controls were recruited from the Affiliated Brain Hospital of Nanjing Medical University from May 2015 to February 2019. HCs were selected by matching patients in gender and age. Clinical diagnosis was performed by two independent neurology experts, using McKeith's criteria for probable DLB (1). All patients suffered from two or more core DLB symptoms. Motor symptoms were evaluated by the Unified Parkinson's Disease Rating Scale-Part III (UPDRS-III) and the severity of disease was obtained by the Hoehn and Yahr (H-Y) score. All participants underwent cognitive condition assessments by the Mini-Mental State Examination (MMSE) and Montreal Cognitive Assessment (MoCA). Subjects who had MRI contraindications, a history of focal brain lesions, other neurological or psychiatric diseases, and severe medical illness were excluded from both groups. The study was approved by the Human Participants Ethics Committee of the Affiliated Brain Hospital of Nanjing Medical University, China, and each subject enrolled in this study provided written informed consent.

### Image Acquisition

All MRI scans were obtained from a 3.0 Tesla MRI scanner (Siemens, Verio, Germany) at the Department of Radiology of the Affiliated Brain Hospital of Nanjing Medical University, Nanjing, China. Resting-state functional images were collected by an 8-channel head coil and a gradient-echo T2-weighted echo planar imaging (EPI) sequence according to the following parameters: resolution = 3 × 3 × 3 mm^3^; time points = 140; repetition time (TR) = 2,000 ms; echo time (TE) = 30 ms; field of view (FOV) = 240 × 240 mm; flip angle = 90°; matrix = 64 × 64; slice number = 30; thickness = 3 mm; slice gap = 0 mm. High-resolution T1-weighted images were acquired using three-dimensional fast spoiled gradient-echo (3D FSPGR) sequence in a sagittal orientation for each subject (resolution = 1 × 1 × 1 mm^3^; TR = 2530 ms; TE = 3.34 ms; flip angle = 7°; slice number = 128; thickness = 1.33 mm; slice gap = 0 mm; matrix = 256 × 256). During the scanning, participants were explicitly instructed to be relaxed, to close their eyes, to not think of anything, and to remain still and awake (confirmed by all subjects immediately after the scanning). Additionally, the head of all participants was fixed and earplugs were used in order to reduce the impact of head motion and noise.

### Data Processing

Image preprocessing was performed using Data Processing & Analysis for (Resting-State) Brain Imaging (DPABI Version 2.3 http://rfmri.org/dpabi) implemented in MATLAB 2014a (MathWorks, Natick, MA). The preprocessing steps were as follows: reduction of the first 10 volumes of each rest session, slice timing, realignment to the middle image for head motion correction, registration to mean functional image; spatial normalization (3 × 3 × 3 mm^3^) to standard space (Montreal Neurological Institute, MNI) using Diffeomorphic Anatomical Registration Through Exponentiated Lie algebra (DARTEL); segmentation of anatomical images into gray matter (GM), white matter (WM), and cerebrospinal fluid (CSF); removal of the WM signals, CSF signals, and Friston 24-parameter; smooth with 6 mm full-width half-maximum (FWHM) kernel, regressing out linear and quadratic trends and band-pass filter (0.01–0.08 Hz). This preprocessing order was in accordance with the standard protocol as described in Yan et al. (22). Subjects with head motion >2.0° and 2 mm were excluded.

### ROI Generation and Definition of Functional Brain Networks

The complexity of time-varying DFC represents the main difference from static FC and the recruitment of more areas in the relevant networks could offer more comprehensive information. As shown in a previous study (23), after preprocessing, the 264 spherical regions of interest (ROIs) with a diameter of 5 mm were defined around coordinates defined by Power et al. (24). Then, the mean time courses of the 264 ROIs were extracted. According to previous studies, these functional regions form 13 brain networks, including DMN, SMN, cingulo-opercular network (CON), auditory network, VIS, memory retrieval, FPN, salience network (SAN), subcortical network (SC), ventral attention network (VAN), dorsal attention network (DAN), cerebellum network, and uncertain networks. This definition of brain network facilitates the study of FC within and between networks. Since the function of the “Uncertain” network is not specific, we mainly paid attention to the other 12 brain networks consisting of 236 seed sites.

### Dynamic Functional Network Connectivity Construction

The 236 available ROIs were used to analyze DFC using DynamicBC toolbox (www.restfmri.net/forum/DynamicBC). Since there was no formal consensus on window length, dynamic functional network connectivity (dFNC) was constructed using the sliding-window Pearson's correlation method with a length of 20 TRs (40 s) and a step size of 1TR, as previously performed (25), resulting in 111 windows. In each window, we computed Pearson correlation coefficients between the time series of each pair of the 236 ROIs. As a result, we obtained a 236 × 236 matrix of Pearson correlation coefficients between any pair of ROIs to construct the brain network FC matrix for each window. Then, Fisher's *r*-to-*z* transformation was used to transform the unweighted individual correlation matrices into *z*-score matrices without thresholding, so as to improve normality. These Fisher's *z*-transformed correlation matrices were used to further calculate the variation of DFC. The static functional network connectivity was also calculated based on the whole time series.

### Functional Connectivity Variation

To characterize dynamics variability, the dynamic functional connectivity variation (dFCV) matrix was subsequently evaluated as the standard deviation of the Fisher's *z*-transformed Pearson's correlation coefficients across all of the windows for each participant. In this method, DFC stability could be quantitatively estimated and compared between different groups. The original FCV matrices were then Fisher *z*-transformed and were statistically compared.

### *k*-means Clustering

To identify dFNC patterns reoccurring across temporal matrices, *k*-means clustering was employed on all the dynamic correlation matrices to divide the dFNC into discrete clusters. The *k*-means algorithm aggregates information with similarities into “*k*” groups, ensuring that the sum of squares within clusters is minimal. During the clustering estimation, the correlation distance metric was chosen because it is more sensitive to the dFNC pattern, regardless of magnitude (26). Cluster analysis of a series of *k* values ranging from 2 to 10 was then performed based on all subjects. To obtain the optimal number of cluster centroids, we used the silhouette values, Calinski–Harabasz values and Davies–Bouldin values. The evaluation results showed that (silhouette) optimal *k* = 2, (Calinski–Harabasz) optimal *k* = 2, (Davies Bouldin) optimal *k* = 2, and (mean) optimal *k* = 2, where (mean) optimal *k* = 2 is the value obtained by rounding the first three (2, 2, 2) simple average backwards. Finally, we gave the result of optimal *k* = 2. For each available cluster, a cluster centroid was generated, representing the FC for this cluster capturing all features. Therefore, two most frequent states of DFC were identified and further statistical analysis was carried out between patients and HC.

### State Analysis

The temporal indexes derived from each participant's transition state vector, which represents the change of state assignments over time, were explored. More specifically, we focus on three main measures of each subject, including (a) mean dwell time of each state, which refers to the average number of consecutive windows belonging to one state before changing to the other state; (b) frequency of each state, which refers to the number of windows per state; and (c) total number of transitions, which refers to the total number of conditions that the clustering state convert from one to another.

### Topological Properties of Static Functional Connectivity

To better understand the disease effects on the resting state fMRI, graph metrics for the static connectivity were also computed. Individual correlation matrices were converted into binarized matrices according to a predefined sparsity threshold (0.01–0.50, with an increment of 0.01). Network sparsity, defined as the fraction of the total number of existing edges divided by the maximum possible number of edges, was used to enable all networks to have the same number of edges. This threshold selection was based on the criteria proposed by previous studies (27, 28), which could assure that the thresholded networks were estimable for small-worldness and had sparse properties with as few spurious edges as possible. Global efficiency and local efficiency, which are two key topological properties, were employed using the graph theoretical network analysis toolbox. Global efficiency computed the average inverse shortest path length of all network nodes, as a measure of global integration, quantifying the parallel information transfer (29). Local efficiency estimated the information exchange capability of subnetwork, as an assessment of local connection efficiency, measuring the fault tolerance of networks (29). Since each correlation matrix was thresholded repeatedly over a wide range of sparsity, the area under the curve (AUC) for each network metric was computed to be taken as a summarized scalar for the topological characterization of the brain networks. The statistical analysis was performed between DLB and HC subjects to compare the static network features.

### Statistical Analysis

The Fisher *z*-transformed FCV matrices were compared between patients and HC using two-sample *t*-test using the graph theoretical network analysis toolbox (*p* < 0.005). Analyses were performed using age and sex as covariates. Since data distribution was not normal, non-parametric Mann–Whitney test was used to compare the dwell time of each state, frequency of each state and total number of transitions between patients and controls using a threshold of *p* < 0.05. Besides, statistical analysis to evaluate local efficiency, global efficiency, age and clinical assessment was conducted using two sample *t*-test, while statistical analysis to evaluate significant differences in the number of males and females between the two groups was conducted applying the Chi-square test.

## Results

### Demographic and Clinical Information of the Subjects

Demographic and clinical information of the participants is summarized in Table 1. No significant differences were found in gender and age between patients and HC. As expected, the MMSE and MoCA scores were lower in the DLB group (*p* = 0.000).

**Table 1:** Demographic and clinical information of the subjects.

	**DLB (*n* = 17)**	**HC (*n* = 20)**	**χ2/*T***	***p*-value**
Gender (male/female)	13/4	9/11	3.776^a^	0.052
Age (years)	71.88 (5.69)	67.80 (7.19)	1.891^b^	0.067
MMSE	19.06 (4.81)^*^	29.05 (0.89)	8.435^b^	0.000
MoCA	13.35 (5.44)^*^	27.20 (1.15)	10.297^b^	0.000
Disease duration (years)	2.68 (1.57)	—	—	—
Hoehn and Yahr (H–Y) score	2.50 (1.10)	—	—	—
UPDRS-III (motor)	17.06 (7.66)	—	—	—

Numbers are expressed as mean (standard deviation). The asterisk (^*^) represents significant inter-group differences.

a *χ2 value of chi-square test*.

b *t value of two-sample t tests*.

*DLB, Dementia with Lewy body; HC, healthy control; MMSE, Mini Mental State Examination; MoCA, Montreal Cognitive Assessment; UPDRS-III, Unified Parkinson's Disease Rating Scale-Part III*.

### FCV

**Figure 1:**
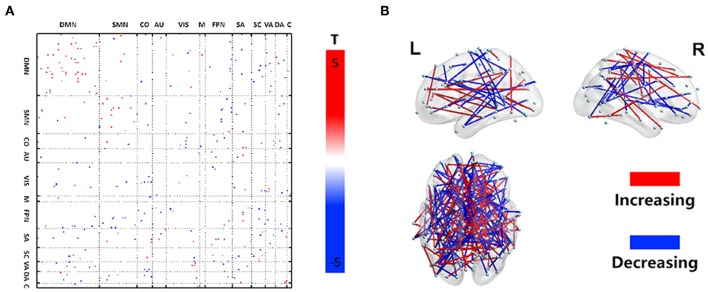
Dynamic functional network connectivity characteristics. Illustration of the statistical analysis results of the dFCV between DLB and HC. **(A)** Depicts the dFCV difference matrix. Red dots represent an increase of variation in the DLB group, while blue dots represent a decrease of variation in the DLB group. **(B)** A 3D visualization of the results in **(A)** (*p* < 0.005).

Disease-related alterations in dFCV compared with the HC are shown in Figures 1A,B. All changes in the DLB group indicated that most of the increased DFC variations are located within the networks (DMN, SMN, VIS, FPN, SA, SC, and DA), and few intra-network FCV showed a decrease within the DMN and SC. Inter-network changes were characterized by decreased DFC variations, especially between several subnetwork pairs related to DMN, VIS, and FPN, and between SMN and FPN. Besides, some subnetwork regions exhibited mixed changes including both increase and decrease.

### State Connectivity Patterns and Clustering Indexes

**Figure 2:**
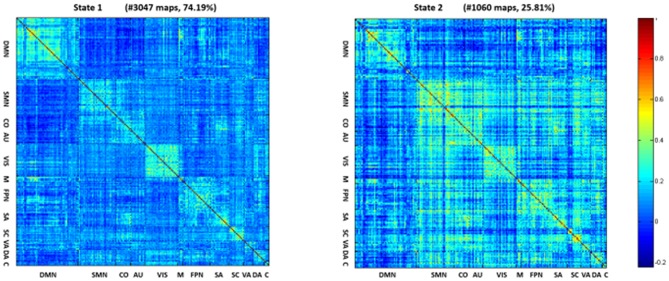
Centroids of the two dFNC states. In state 1, the whole network displayed slight negative connectivity and slight positive connectivity intra-networks. State 2 showed moderate positive connectivity within and between most inter-networks, while the connectivity was negative between DMN and others.

**Figure 3:**
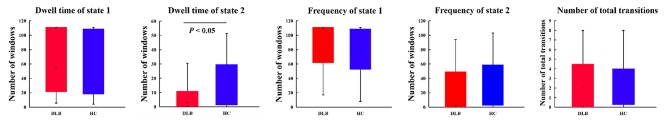
Clustering index characteristics. Group comparison of state dwell time, occurrence frequency, and number of total transitions. The upper and lower boundaries of the box-plot represent the maximum and minimum values, the upper and lower boundaries of the box body represent the upper and lower quartiles, respectively, and the short line in the box body represents the median. *p*-value results from Mann–Whitney test. See Table 2 for detailed information on statistics.

The (mean) optimal value of *k* is 2. Thus, two representative states were identified for all obtained sliding windows from the clustering analysis and the centroids of the two dFNC states, indicating the median of all subjects for each state, as shown in Figure 2. The percentages located at the top of each centroid represent the occurrence of each state to total time series. In state 1, the whole network displayed slight negative connectivity, and slight positive connectivity intra-networks. State 2 showed moderate positive connectivity within and between most inter-networks, while the connectivity was negative between DMN and others. Additionally, based on the *k*-means algorithm results, the dwell time and frequency of each state and the total number of transitions were characterized, and the relevant indexes of connectivity states are shown in Figure 3 (Table 2). Inter-group differences observed in the dwell time revealed that the average dwell time of state 2 in the DLB group was significantly shortened compared with normal volunteers (*p* = 0.040). Although no significant difference in dwell time of state 1 or total transition times between the two groups was found, the DLB patients showed an increasing trend in state 1 dwell time and a decreasing trend in the total transition time.

**Table 2:** Clustering indexes of each group.

	**DLB (*n* = 17)**	**HC (*n* = 20)**	***U*^**a**^**	***p-*value**
Dwell time of state 1	54 (21.38, 111)	33.08 (17.79, 108.75)	126.00	0.170
Dwell time of state 2	3 (0, 10.90)^*^	11.33 (1.38, 29.75)	104.00	0.040
Frequency of state 1	108 (61.5, 111)	79.5 (52.25, 108.75)	123.00	0.143
Frequency of state 2	3 (0, 49.5)	31.5 (2.25, 58.75)	123.00	0.143
Number of total transitions	2 (0, 4.5)	3 (0.25, 4)	142.00	0.380

*Numbers are expressed as median value (Percent 25, Percent 75)*.

a U value of the Mann–Whitney test. The asterisk (^*^) represents significant inter-group differences.

### Topological Properties of Static Functional Connectivity

**Figure 4:**
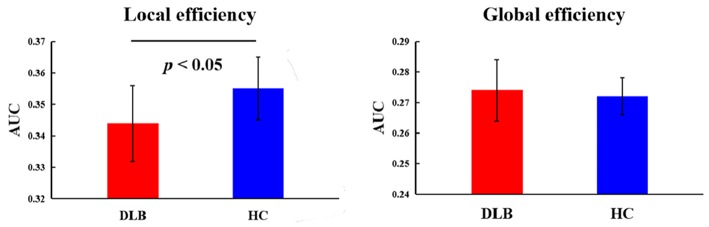
Topological properties of static functional connectivity. The comparison between HC and the DLB group with respect to the local and global efficiency. *p*-value results from two-sample *t*-tests. See Table 3 for detailed information.

Using the graph theory analysis, significant differences between groups were observed in the local efficiency (Table 3, Figure 4). The DLB group had a significantly lower local efficiency than HC, whereas no significant findings were obtained regarding global efficiency.

**Table 3:** Static brain network properties of each group.

	**DLB (*n* = 17)**	**HC (*n* = 20)**	**Test statistics^**a**^**	***p*-value**
Global efficiency	0.274 (0.010)	0.272 (0.006)	0.893	0.378
Local efficiency	0.344 (0.012)^*^	0.355 (0.010)	3.074	0.004

*Numbers are expressed as mean (standard deviation)*.

a t value of two-sample t-tests. The asterisk (^*^) represents significant inter-group differences. DLB, Dementia with Lewy body; HC, healthy control.

## Discussion

In the present study, the features of the dynamic time-varying FC in DLB patients were explored in view of a large-scale brain network derived from rs-fMRI using the sliding-window method. Our observations primarily demonstrated that the dFCV matrix of the DLB group presented a mixed change characteristic. Specifically, DLB patients displayed increased intra-network variability mainly involving DMN, SMN, VIS, FPN, and decreased dFCV between DMN, VIS, FPN, and between SMN and FPN. Subsequently, *k*-means algorithm identified two representative states among all subjects, and the dwell time of state 2 in DLB group was significantly decreased compared to healthy volunteers. Furthermore, the evidence provided by the supplementary graph theory analysis showed that the topological property of DLB was affected with lower local efficiency when compared with healthy volunteers. These findings suggest that the DLB group was characterized by a widely aberrant dFCV and altered functional connection patterns, thus offering a new perspective and ideas in understanding DLB.

### Distribution of dFCV Changes in DLB Patients

Evidence was found regarding some pathophysiological activities of nerve cells associated with the changes of FC. These findings could reflect the intrinsic mechanism under different conditions. The dFCV of DLB patients increased within networks and decreased between networks compared with the normal control group, the former group showing different profiles of intra- and inter-networks. The intra- and inter-network variability characterizes the alterations with time of functional connectivity within and between brain networks. Generally speaking, less fluctuation of variability suggests a higher correlation of function construction among all sliding windows, signifying a lack of flexibility to change. Significant fluctuation of variability implies that the function construction is poorly correlated among different sliding windows, indicating an unstable state (30). Dramatic increase or decrease in dFCV may be associated with potentially pathological neuropsychiatric activities related to cognitive activities (31, 32), mental activities (26, 33), and sensorimotor activities (34). Our results indicated a significantly higher instability within network connectivity and significant lower flexibility between network connectivity in DLB patients, which reflects distinct patterns of functional intra- and inter-network damage, deepening our understanding on the pathological mechanism of DLB.

Normally, each network module is equipped with specific functions, and the functional connection between network modules can be understood as the organic integration of different functions. Any abnormal function of either within and between brain network will affect the normal execution of specific functions undertaken by brain networks, leading to clinical symptoms in DLB patients. The increase of dynamic FCV within DMN, SMN, VIS, and FPN in the DLB group implies that the connection state is aberrant and more unstable. DMN is related to cognitive processing including episodic memory and making plans (35). VIS is associated to visual perception (36) and visual-constructive processing (37). FPN is responsible for the execution control of attention (38, 39). Since DLB individuals complain mainly about attention (40), motor (2), and visual (40) impairments and these dysfunctions are roughly consistent with the functions of DMN, SMN, VIS, and FPN, respectively, we therefore speculate that the occurrence of these symptoms could be related to the functional instability of the networks mentioned above. Besides, these abnormal networks and the brain regions involved were also reported in previous imaging studies of DLB. Metabolism (41) and perfusion (42, 43) studies found a decrease of neural activities in the frontal, parietal, and occipital cortex areas. A large number of fMRI studies reported a disrupted functional connectivity of DMN (5, 6), SMN (4), VIS (5), and FPN (4, 8) in DLB patients. These studies further support the reliability of our findings. Therefore, we have reason to believe that these unstable intra-network connections could provide a theoretical support for transient clinical features such as spontaneous fluctuation in attention, cognition, and recurrent visual hallucinations in DLB patients.

From the perspective of interconnection, large-scale brain network pairs with a lower variability, encompassing DMN, VIS, FPN and SMN, cannot be flexibly integrated, which may be related to the poor cognitive, motor, visual, sleep, and emotional impairments in DLB patients in general, resulting in a complex treatment strategy and no ideal therapeutic effect. Furthermore, previous works have been carried out to illustrate the neural basis of DLB, with substantial evidence suggesting widespread WM lesions (44, 45) and structural atrophy (46). Besides, a previous work indicated inconsistent regional homogeneity of neural activities (47) and an abnormal graph model of information communication between distant and close brain regions in DLB participants (9). A large number of structural damage and functional changes may be the neural basis of dysfunctional dynamics of intra- and inter-network connectivity in DLB. Taken together, these inter-group differences within and between networks consistent with clinical impairments suggest that the temporal dynamic characteristics of brain networks could reflect DLB dysfunction-related neural phenomena.

### Clustering Indexes Alterations in DLB Participants

DFC study covering the whole brain in non-task state (48) revealed that the human brain is in a dynamic transition process between diverse FCs, which are characterized by different connectivity patterns (49) (commonly referred to as “state”). The dynamic change of FC underlined that the organization reconstruction takes place constantly, creating highly flexible and adaptable systems without fundamentally structural alteration (50, 51) and some recurring patterns can be finally identified (52). These patterns may undergo changes with internal external drivers, ranging from physiology (52, 53) to pathology. For instance, with the development of DLB, the frequency, duration, and flexible switching between distinct states may be affected in the patient group.

In the current study, we captured the inter-group differences in the dwell time of state 2. The results showed a significantly fewer state 2 average dwell time in the DLB group, together with a trend of lower frequency. In addition, we provided evidence that the DLB group had the tendency of longer dwell time and higher occurrence frequency of state 1. In state 2, almost all intra-networks and most inter-networks displayed moderate positive connectivity, while DMN displayed a negative connectivity pattern between networks. This connectivity pattern may signify that the subjects are on the alert to gather information from their surroundings (54). In state 1, the whole network displayed a slight negative connectivity, with slight positive connectivity located within networks. This pattern may mean that the participants reduced the focus on the outside and are in a resting state (54). DLB individuals allocate less time to a state of positive connectivity and devote more time to negative connectivity pattern than controls, which might suggest that brain network connectivity of the DLB tends to be segregated, while the brain network connectivity of the control might prefer to be integrated during resting state. This may be relevant to the presence of cognitive decline in general.

State conversions could be the foundation of segregation and integration between different brain networks/subnetworks or specific functional resources such as cognitive (55, 56) and mental activities (26). As expected, DLB individuals switched less frequently, which might indicate less response to the surrounding information and might result in a slow thinking and intermittent confusion, which are typical DLB features. Overall, the observed slowing and disturbance of state dynamics in DLB suggest that the brain of DLB patients might be in a less flexible and ineffective functional state.

### Topological Characteristics of Resting Brain Network

DFC measures brain network properties well from a temporal perspective. In order to comprehensively depict brain network characteristics, we further complementarily analyzed the brain network properties of spatial topology and found that the local efficiency of the DLB group was significantly lower than that of healthy controls. Local efficiency is an assessment of local information exchange, measuring the fault tolerance of networks. This supplementary analysis proved from another point of view that DLB patients had abnormal network function. Time-based DFC analysis revealed a loss of brain network flexibility in DLB patients. From the perspective of space, we found that the local fault tolerance in DLB patients was reduced. Reduced fault tolerance means that the brain is less able to withstand failure and recover from failure, which might be associated with DLB's abnormally rigid brain networks, thus leading to the cognitive slowing. Interestingly, we found that the global efficacy of DLB is normal, suggesting that these patients only had abnormal local indicators while global indicators were not affected, which may be related to functional compensation driven from mixed changed within and between networks in respect of dynamic FCV.

## Limitations

Several limitations of our study should be considered. First, our sample size was relatively small and the statistical effect was consequently reduced, resulting in imaging results not passing multiple comparative corrections. Nevertheless, efforts are currently made to recruit more subjects, meaning that a larger sample size will be used in our further study to prove the validity of these findings. Second, DLB patients in our study received medication and the potential impact of drugs on nervous activity is difficult to be removed. However, dopaminergic drugs have been proved to normalize brain functional connections (57), implying that the statistical differences between groups are not drug-related. Besides, a previous work reported that the administration of memantine resulted in an increased FC in Alzheimer's disease subjects (58), but this drug was not administered to any of the DLB patients in our cohort. In addition, there are a variety of analytical approaches for calculating matrices and make inference on their significance. Based on this, we used the commonly accepted methods, including using Fisher *z* transformation to improve the normality and calculation of the AUC of each network metric as the sum scalar of the topological characteristics of brain network to prove the reliability of the results. Even if some output results could not be fully explained, a data-driven model based on extracting time series of brain networks for each DLB subject could be considered as a promising research method for describing the dynamics of resting state networks. The development of image analysis and the combination of multimodal techniques may be more helpful to reveal the disease characteristics accurately and comprehensively.

## Conclusion

Our exploratory experiments combining sliding-window and clustering methods analyzed the dynamics of functional network connectivity. In this work an increased intra-network variability and decreased inter-network in DLB was reported, clearly distinguishing DLB from HC and which might be associated with transient clinical features such as fluctuating cognition and recurrent visual hallucinations. Disturbances to the time allocation of state sequences and a less dynamic brain in DLB lead to a loss of integration and flexibility of the brain network, which might be related to symptoms such as slow thinking and intermittent confusion. Aberrant brain network dynamics of DLB could be considered as a neuroimaging marker of various clinical manifestations. The current study proves the power of the dynamic neuroscience method in the exploration of brain network activity of neurodegenerative diseases and deepens our understanding of the pathophysiological mechanisms in DLB.

## Data Availability Statement

The datasets generated for this study are available on request to the corresponding author.

## Ethics Statement

The studies involving human participants were reviewed and approved by Human Participants Ethics Committee of the Affiliated Brain Hospital of Nanjing Medical University, China. The patients/participants provided their written informed consent to participate in this study.

## Author Contributions

J-PS and JC designed the study and revised it critically for important content. W-YM performed the research and drafted the manuscript. QY and GH helped in data analyses. C-YX helped in clinical data collection and analyses.

### Conflict of Interest

The authors declare that the research was conducted in the absence of any commercial or financial relationships that could be construed as a potential conflict of interest.

## References

[1] McKeith IG Dickson DW Lowe J Emre M O'Brien JT Feldman H. Diagnosis and management of dementia with Lewy bodies: third report of the DLB Consortium. Neurology. (2005) 65:1863–72. 10.1212/01.wnl.0000187889.17253.b1 16237129

[2] McKeith IG Boeve BF Dickson DW Halliday G Taylor JP Weintraub D. Diagnosis and management of dementia with Lewy bodies: Fourth consensus report of the DLB Consortium. Neurology. (2017) 89:88–100. 10.1212/WNL.0000000000004058 28592453 PMC5496518

[3] Kenny ER O'Brien JT Firbank MJ Blamire AM. Subcortical connectivity in dementia with Lewy bodies and Alzheimer's disease. Br J Psychiatry. (2013) 203:209–14. 10.1192/bjp.bp.112.108464 23846997

[4] Peraza LR Colloby SJ Firbank MJ Greasy GS McKeith IG Kaiser M. Resting state in Parkinson's disease dementia and dementia with Lewy bodies: commonalities and differences. Int J Geriatr Psychiatry. (2015) 30:1135–46. 10.1002/gps.4342 26270627 PMC4737212

[5] Galvin JE Price JL Yan Z Morris JC Sheline YI. Resting bold fMRI differentiates dementia with Lewy bodies vs Alzheimer disease. Neurology. (2011) 76:1797–803. 10.1212/WNL.0b013e31821ccc83 21525427 PMC3100121

[6] Lowther ER O'Brien JT Firbank MJ Blamire AM. Lewy body compared with Alzheimer dementia is associated with decreased functional connectivity in resting state networks. Psychiatry Res. (2014) 223:192–201. 10.1016/j.pscychresns.2014.06.004 25035300

[7] Schumacher J Peraza L R. Functional connectivity in dementia with Lewy bodies: a within- and between-network analysis. Hum Brain Mapp. (2018) 39:1118–29. 10.1002/hbm.23901 29193464 PMC5900719

[8] Peraza LR Kaiser M Firbank M Graziadio S Bonanni L Onofrj M. fMRI resting state networks and their association with cognitive fluctuations in dementia with Lewy bodies. Neuroimage Clin. (2014) 4:558–65. 10.1016/j.nicl.2014.03.013 24818081 PMC3984441

[9] Peraza LR Taylor JP Kaiser M. Divergent brain functional network alterations in dementia with Lewy bodies and Alzheimer's disease. Neurobiol Aging. (2015) 36:2458–67. 10.1016/j.neurobiolaging.2015.05.015 26115566 PMC4706129

[10] Handwerker DA Roopchansingh V Gonzalez-Castillo J Bandettini PA. Periodic changes in fMRI connectivity. Neuroimage. (2012) 63:1712–9. 10.1016/j.neuroimage.2012.06.078 22796990 PMC4180175

[11] Zuo XN Xu T Jiang L Yang Z Cao XY He Y. Toward reliable characterization of functional homogeneity in the human brain: preprocessing, scan duration, imaging resolution and computational space. Neuroimage. (2013) 65:374–86. 10.1016/j.neuroimage.2012.10.017 23085497 PMC3609711

[12] Chang C Glover GH. Time-frequency dynamics of resting-state brain connectivity measured with fMRI. Neuroimage. (2010) 50:81–98. 10.1016/j.neuroimage.2009.12.011 20006716 PMC2827259

[13] Calhoun VD Miller R Pearlson G Adali T. The chronnectome: time-varying connectivity networks as the next frontier in fMRI data discovery. Neuron. (2014) 84:262–74. 10.1016/j.neuron.2014.10.015 25374354 PMC4372723

[14] Hutchison RM Womelsdorf T Gati JS Everling S Menon RS. Resting-state networks show dynamic functional connectivity in awake humans and anesthetized macaques. Hum Brain Mapp. (2013) 34:2154–77. 10.1002/hbm.22058 22438275 PMC6870538

[15] Grana M Ozaeta L Chyzhyk D. Resting state effective connectivity allows auditory hallucination discrimination. Int J Neural Syst. (2017) 27:1750019. 10.1142/S0129065717500198 28274168

[16] Demirtas M Tornador C Falcon C Lopez-Sola M Hernandez-Ribas R Pujol J. Dynamic functional connectivity reveals altered variability in functional connectivity among patients with major depressive disorder. Hum Brain Mapp. (2016) 37:2918–30. 10.1002/hbm.23215 27120982 PMC5074271

[17] Rashid B Damaraju E Pearlson GD Calhoun VD. Dynamic connectivity states estimated from resting fMRI Identify differences among Schizophrenia, bipolar disorder, and healthy control subjects. Front Hum Neurosci. (2014) 8:897. 10.3389/fnhum.2014.00897 25426048 PMC4224100

[18] Du Y Pearlson GD Yu Q He H Lin D Sui J. Interaction among subsystems within default mode network diminished in schizophrenia patients: a dynamic connectivity approach. Schizophr Res. (2016) 170:55–65. 10.1016/j.schres.2015.11.021 26654933 PMC4707124

[19] Liu F Wang Y Li M Wang W Li R Zhang Z. Dynamic functional network connectivity in idiopathic generalized epilepsy with generalized tonic-clonic seizure. Hum Brain Mapp. (2017) 38:957–73. 10.1002/hbm.23430 27726245 PMC6866949

[20] Wee CY Yang S Yap PT Shen D. Sparse temporally dynamic resting-state functional connectivity networks for early MCI identification. Brain Imaging Behav. (2016) 10:342–56. 10.1007/s11682-015-9408-2 26123390 PMC4692725

[21] Sourty M Thoraval L Roquet D Armspach JP Foucher J Blanc F. Identifying dynamic functional connectivity changes in dementia with Lewy bodies based on product hidden Markov models. Front Comput Neurosci. (2016) 10:60. 10.3389/fncom.2016.00060 27445778 PMC4918689

[22] Yan CG Wang XD Zuo XN Zang YF. DPABI: data processing & analysis for (resting-state) brain imaging. Neuroinformatics. (2016) 14:339–51. 10.1007/s12021-016-9299-4 27075850

[23] Braun U Schafer A Walter H Erk S Romanczuk-Seiferth N Haddad L. Dynamic reconfiguration of frontal brain networks during executive cognition in humans. Proc Natl Acad Sci USA. (2015) 112:11678–83. 10.1073/pnas.1422487112 26324898 PMC4577153

[24] Power JD Cohen AL Nelson SM Wig GS Barnes KA Church JA. Functional network organization of the human brain. Neuron. (2011) 72:665–78. 10.1016/j.neuron.2011.09.006 22099467 PMC3222858

[25] Lin SJ Vavasour I Kosaka B Li DKB Traboulsee A MacKay A. Education, and the balance between dynamic and stationary functional connectivity jointly support executive functions in relapsing-remitting multiple sclerosis. Hum Brain Mapp. (2018) 39:5039–49. 10.1002/hbm.24343 30240533 PMC6866468

[26] Zhang W Li S Wang X Gong Y Yao L Xiao Y. Abnormal dynamic functional connectivity between speech and auditory areas in schizophrenia patients with auditory hallucinations. Neuroimage Clin. (2018) 19:918–24. 10.1016/j.nicl.2018.06.018 30003029 PMC6039841

[27] Lei D Li K Li L Chen F Huang X Lui S. Disrupted functional brain connectome in patients with posttraumatic stress disorder. Radiology. (2015) 276:818–27. 10.1148/radiol.15141700 25848901

[28] Zhang Z Liao W Chen H Mantini D Ding JR Xu Q. Altered functional-structural coupling of large-scale brain networks in idiopathic generalized epilepsy. Brain. (2011) 134(Pt 10):2912–28. 10.1093/brain/awr223 21975588

[29] Latora V Marchiori M. Efficient behavior of small-world networks. Phys Rev Lett. 87:198701. 10.1103/PhysRevLett.87.198701 11690461

[30] Zhu H Huang J Deng L He N Cheng L Shu P. Abnormal dynamic functional connectivity associated with subcortical networks in parkinson's disease: a temporal variability perspective. Front Neurosci. 13:80. 10.3389/fnins.2019.00080 30837825 PMC6389716

[31] Quevenco FC Preti MG van Bergen JM Hua J Wyss M Li X. Memory performance-related dynamic brain connectivity indicates pathological burden and genetic risk for Alzheimer's disease. Alzheimers Res Ther. 9:24. 10.1186/s13195-017-0249-7 28359293 PMC5374623

[32] Viviano RP Raz N Yuan P Damoiseaux JS. Associations between dynamic functional connectivity and age, metabolic risk, and cognitive performance. Neurobiol Aging. (2017) 59:135–43. 10.1016/j.neurobiolaging.2017.08.003 28882422 PMC5679403

[33] Du Y Fryer SL Fu Z Lin D Sui J Chen J. Dynamic functional connectivity impairments in early schizophrenia and clinical high-risk for psychosis. Neuroimage. (2018) 180:632–45. 10.1016/j.neuroimage.2017.10.022 29038030 PMC5899692

[34] Brovelli A Badier JM. Dynamic reconfiguration of visuomotor-related functional connectivity networks. J Neurosci. (2017) 37:839–53. 10.1523/JNEUROSCI.1672-16.2017 28123020 PMC6597020

[35] Buckner RL Andrews-Hanna JR Schacter DL. The brain's default network: anatomy, function, and relevance to disease. Ann N Y Acad Sci. (2008) 1124:1–38. 10.1196/annals.1440.011 18400922

[36] Beckmann CF DeLuca M Devlin JT Smith SM. Investigations into resting-state connectivity using independent component analysis. Philos Trans R Soc Lond B Biol Sci. (2005) 360:1001–13. 10.1098/rstb.2005.1634 16087444 PMC1854918

[37] Fox MD Corbetta M Snyder AZ Vincent JL Raichle ME. Spontaneous neuronal activity distinguishes human dorsal and ventral attention systems. Proc Natl Acad Sci USA. (2006) 103:10046–51. 10.1073/pnas.0604187103 16788060 PMC1480402

[38] Seeley WW Menon V Schatzberg AF Keller J Glover GH Kenna H. Dissociable intrinsic connectivity networks for salience processing and executive control. J Neurosci. (2007) 27:2349–56. 10.1523/JNEUROSCI.5587-06.2007 17329432 PMC2680293

[39] Markett S Reuter M Montag C Voigt G Lachmann B Rudorf S. Assessing the function of the fronto-parietal attention network: insights from resting-state fMRI and the attentional network test. Hum Brain Mapp. (2014) 35:1700–9. 10.1002/hbm.22285 23670989 PMC6869384

[40] Ferman TJ Smith GE Kantarci K Boeve BF Pankratz VS Dickson DW. Nonamnestic mild cognitive impairment progresses to dementia with Lewy bodies. Neurology. (2013) 81:2032–8. 10.1212/01.wnl.0000436942.55281.47 24212390 PMC3854825

[41] Kantarci K Lowe VJ Boeve BF Weigand SD Senjem ML Przybelski SA. Multimodality imaging characteristics of dementia with Lewy bodies. Neurobiol Aging. (2012) 33:2091–105. 10.1016/j.neurobiolaging.2011.09.024 22018896 PMC3288845

[42] Lobotesis K Fenwick JD Phipps A Ryman A Swann A Ballard C Occipital hypoperfusion on SPECT in dementia with Lewy bodies but not AD. Neurology. (2001) 56:643–9. 10.1212/WNL.56.5.643 11245717

[43] Colloby SJ Fenwick JD Williams ED Paling SM Lobotesis K Ballard C. A comparison of (99m)Tc-HMPAO SPET changes in dementia with Lewy bodies and Alzheimer's disease using statistical parametric mapping. Eur J Nucl Med Mol Imaging. (2002) 29:615–22. 10.1007/s00259-002-0778-5 11976799

[44] Delli Pizzi S Maruotti V Taylor JP Franciotti R Caulo M Tartaro A. Relevance of subcortical visual pathways disruption to visual symptoms in dementia with Lewy bodies. Cortex. (2014) 59:12–21. 10.1016/j.cortex.2014.07.003 25113955

[45] Delli Pizzi S Franciotti R Taylor JP Thomas A Tartaro A Onofrj M. Thalamic involvement in fluctuating cognition in dementia with Lewy bodies: magnetic resonance evidences. Cereb Cortex. (2015) 25:3682–9. 10.1093/cercor/bhu220 25260701 PMC4585510

[46] Sanchez-Castaneda C Rene R Ramirez-Ruiz B Campdelacreu J Gascon J Falcon C. Frontal and associative visual areas related to visual hallucinations in dementia with Lewy bodies and Parkinson's disease with dementia. Mov Disord. (2010) 25:615–22. 10.1002/mds.22873 20175186

[47] Peraza LR Colloby SJ Deboys L O'Brien JT Kaiser M Taylor JP. Regional functional synchronizations in dementia with Lewy bodies and Alzheimer's disease. Int Psychogeriatr. (2016) 28:1143–51. 10.1017/S1041610216000429 26976496 PMC4894061

[48] Hutchison RM Womelsdorf T Allen EA Bandettini PA Calhoun VD Corbetta M. Dynamic functional connectivity: promise, issues, and interpretations. Neuroimage. (2013) 80:360–78. 10.1016/j.neuroimage.2013.05.079 23707587 PMC3807588

[49] Allen EA Damaraju E Plis SM Erhardt EB Eichele T Calhoun VD. Tracking whole-brain connectivity dynamics in the resting state. Cereb Cortex. (2014) 24:663–76. 10.1093/cercor/bhs352 23146964 PMC3920766

[50] Kirschner M Gerhart J. Evolvability. Proc Natl Acad Sci USA. (1998) 95:8420–7. 10.1073/pnas.95.15.8420 9671692 PMC33871

[51] Bassett DS Gazzaniga MS. Understanding complexity in the human brain. Trends Cogn Sci. (2011) 15:200–9. 10.1016/j.tics.2011.03.006 21497128 PMC3170818

[52] Bassett DS Wymbs NF Porter MA Mucha PJ Carlson JM Grafton ST. Dynamic reconfiguration of human brain networks during learning. Proc Natl Acad Sci USA. (2011) 108:7641–6. 10.1073/pnas.1018985108 21502525 PMC3088578

[53] Cole MW Reynolds JR Power JD Repovs G Anticevic A Braver TS. Multi-task connectivity reveals flexible hubs for adaptive task control. Nat Neurosci. (2013) 16:1348–55. 10.1038/nn.3470 23892552 PMC3758404

[54] Xia Y Chen Q Shi L Li M Gong W Chen H. Tracking the dynamic functional connectivity structure of the human brain across the adult lifespan. Hum Brain Mapp. (2019) 40:717–728. 10.1002/hbm.24385 30515914 PMC6865727

[55] Yu Q Erhardt EB Sui J Du Y He H Hjelm D. Assessing dynamic brain graphs of time-varying connectivity in fMRI data: application to healthy controls and patients with schizophrenia. Neuroimage. (2015) 107:345–55. 10.1016/j.neuroimage.2014.12.020 25514514 PMC4300250

[56] Shakil S Lee CH Keilholz SD. Evaluation of sliding window correlation performance for characterizing dynamic functional connectivity and brain states. Neuroimage. (2016) 133:111–28. 10.1016/j.neuroimage.2016.02.074 26952197 PMC4889509

[57] Tahmasian M Bettray LM van Eimeren T Drzezga A Timmermann L Eickhoff CR. A systematic review on the applications of resting-state fMRI in Parkinson's disease: does dopamine replacement therapy play a role? Cortex. (2015) 73:80–105. 10.1016/j.cortex.2015.08.005 26386442

[58] Lorenzi M Beltramello A Mercuri NB Canu E Zoccatelli G Pizzini FB. Effect of memantine on resting state default mode network activity in Alzheimer's disease. Drugs Aging. (2011) 28:205–17. 10.2165/11586440-000000000-00000 21250762

